# Supplementary material for: Complement receptor CD46 co-stimulates optimal human CD8+ T cell effector function via fatty acid metabolism
Source: Nat Commun. 2018 Oct 10;9:4186. doi: 10.1038/s41467-018-06706-z (PMC6180132; doi:10.1038/s41467-018-06706-z)
Supplement: Supplementary file 1 — Supplementary Information [file 41467_2018_6706_MOESM1_ESM.pdf]

SUPPLEMENTARY INFORMATION

Complement receptor CD46 co-stimulates optimal human CD8^{+} T cell effector function via fatty acid metabolism

Arbore et al.

SUPPLEMENTARY FIGURES

Supplementary Figure 1

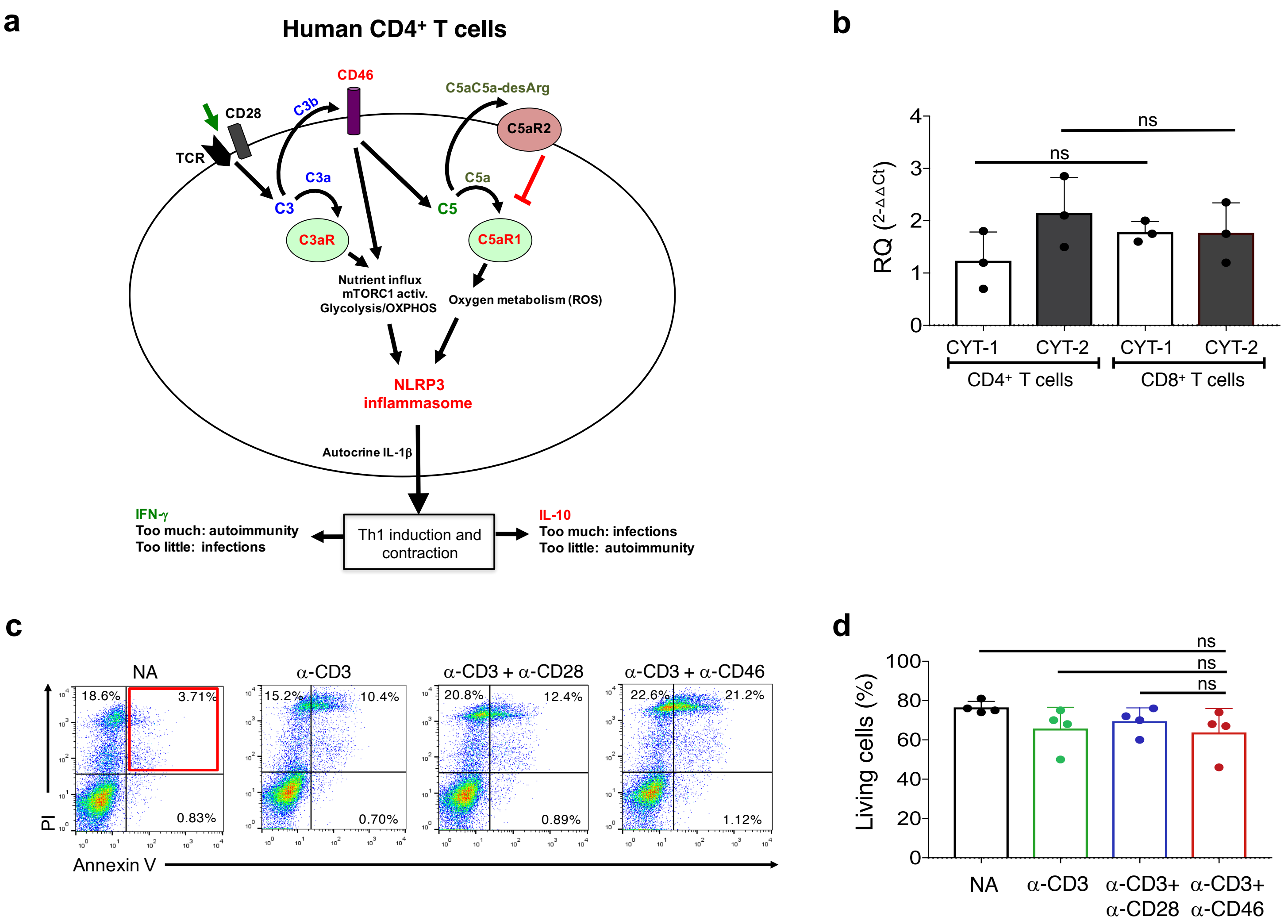

Supplementary Figure 1. CD46 co-stimulation provides superior support for CD8⁺ T cell effector function. **a** Model of complement-regulated inflammasome activation during human Th1 responses: TCR stimulation drives autocrine CD46 engagement and downstream key metabolic events needed specifically for IFN-γ production. CD46 activation also triggers increase in intracellular C5 activation with subsequent intracellular C5aR1-dependent NLRP3 assembly which in turn induces caspase-1-mediated IL-1β maturation. Autocrine IL-1β function promotes IFN-γ production and Th1 induction but restricts 'IL-10 switching'. C5aR2 cell surface activation by secreted C5a (or C5a-desArg) negatively controls these events (either via direct inhibition of C5aR1 activation and/or other yet undefined mechanisms) thereby allowing for IL-10 co-induction during Th1 contraction. **b** CD46 CYT-1 and CYT2 isoform expression mRNA levels in freshly purified non-activated human $CD4^+$ and $CD8^+$ T cells as measured by RT-PCR (n = 3). **c** Representative image of the measurement of $CD8^+$ T cell cytotoxicity upon CD46 costimulation. $CD8^+$ T cells were either left non-activated (NA) or activated for 24 h with Abs to CD3, CD3+CD28 or CD3+CD46 and then seeded onto DU145 cells. After 12 h of co-incubation, cytotoxic activity of T cells towards DU145 cells was determined by measurement of PI and Annexin V staining on DU145 cells (for gating strategy, see Supplementary Fig. 7c). **d** Viability of CD46-activated $CD8^+$ T cells. Cells were either left non-activated or were activated with the depicted immobilized Ab combinations and cell viability assessed at 60 h post activation (n = 4). Error bars denote mean ± SEM. ns, statistically not significant. Statistical analyses were performed using One-way Anova with Tukey Multiple Comparison test or Paired Student's t-test where appropriate.

Supplementary Figure 2

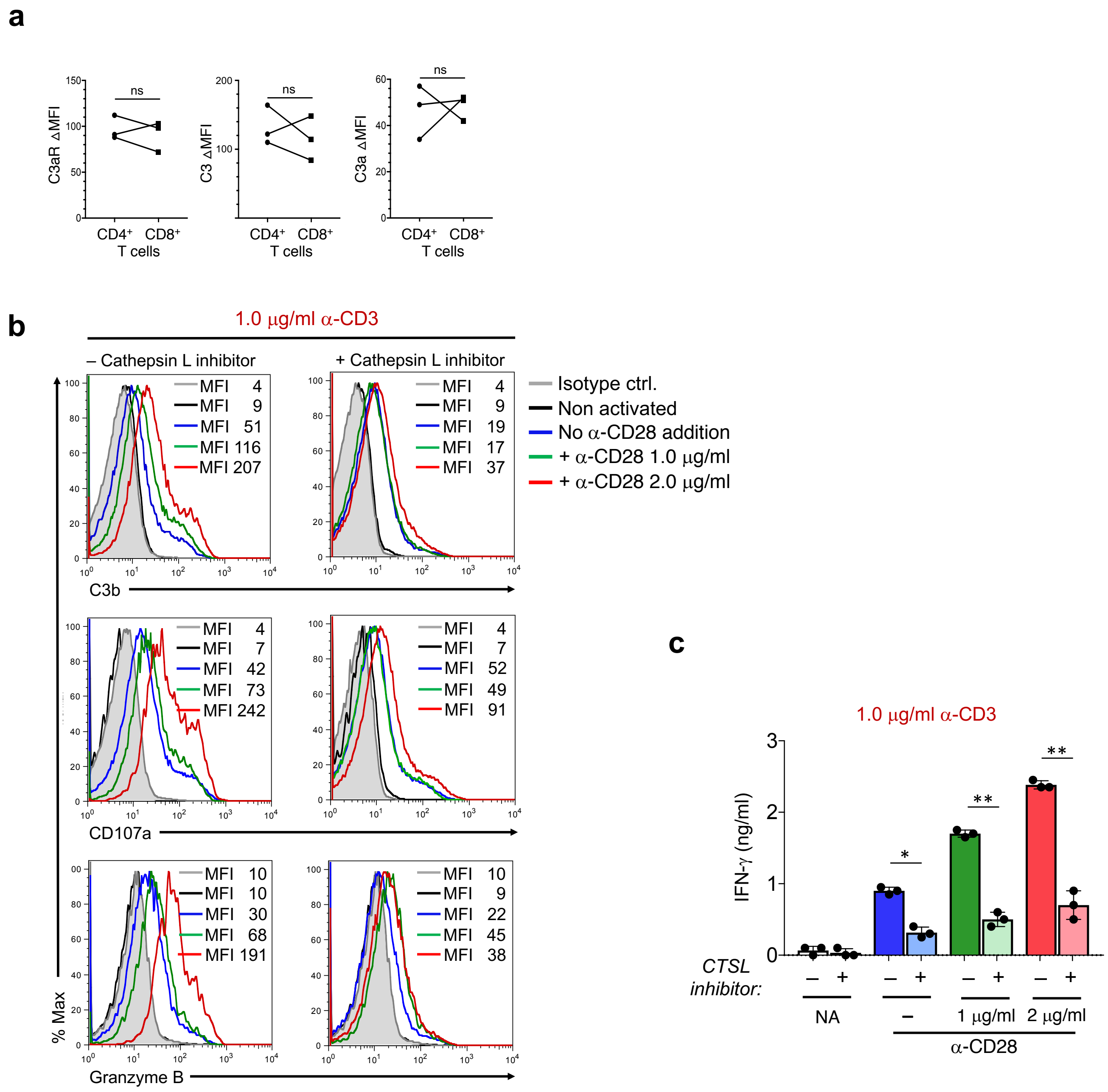

Supplementary Figure 2. TCR and CD28-driven C3b generation supports autocrine CD46 engagement. **a** Detection of intracellular C3aR, complement C3 and the C3 activation fragment C3a in resting human CD4^{+} and CD8^{+} T cells. Freshly purified T cells were permeabilized and assessed via FACS for the presence of C3aR, C3 and C3a (n = 3, gating strategy in Supplementary Fig. 7a). **b** and **c** Effects of cathepsin L inhibition on human CTL activity. Purified human CD8^{+} T cells were activated as depicted in the absence or presence of a cell-permeable cathepsin L inhibitor (50 nM) and surface C3b levels, degranulation and granzyme B expression, and IFN-γ secretion measured at 12 hrs post activation (n = 3). Shown in (**b**) is a representative FACS plot depicting C3b, CD107a, and granzyme B staining (gating strategy in Supplementary Fig. 7b) derived from one of three similarly performed experiments (grey histogram, Isotype control; black histogram, non activated cells; blue, green, and red histograms, 0, 1, and 2 μg/ml α-CD28 addition, respectively) and in (**c**) data summarizing the IFN-γ secretion from three (n = 3) distinct donors. Error bars denote mean ± SEM. *, $p < 0.05$; ns, statistically not significant. CTSL, cathepsin L. Statistical analyses were performed using the Paired Student's t-test.

Supplementary Figure 3

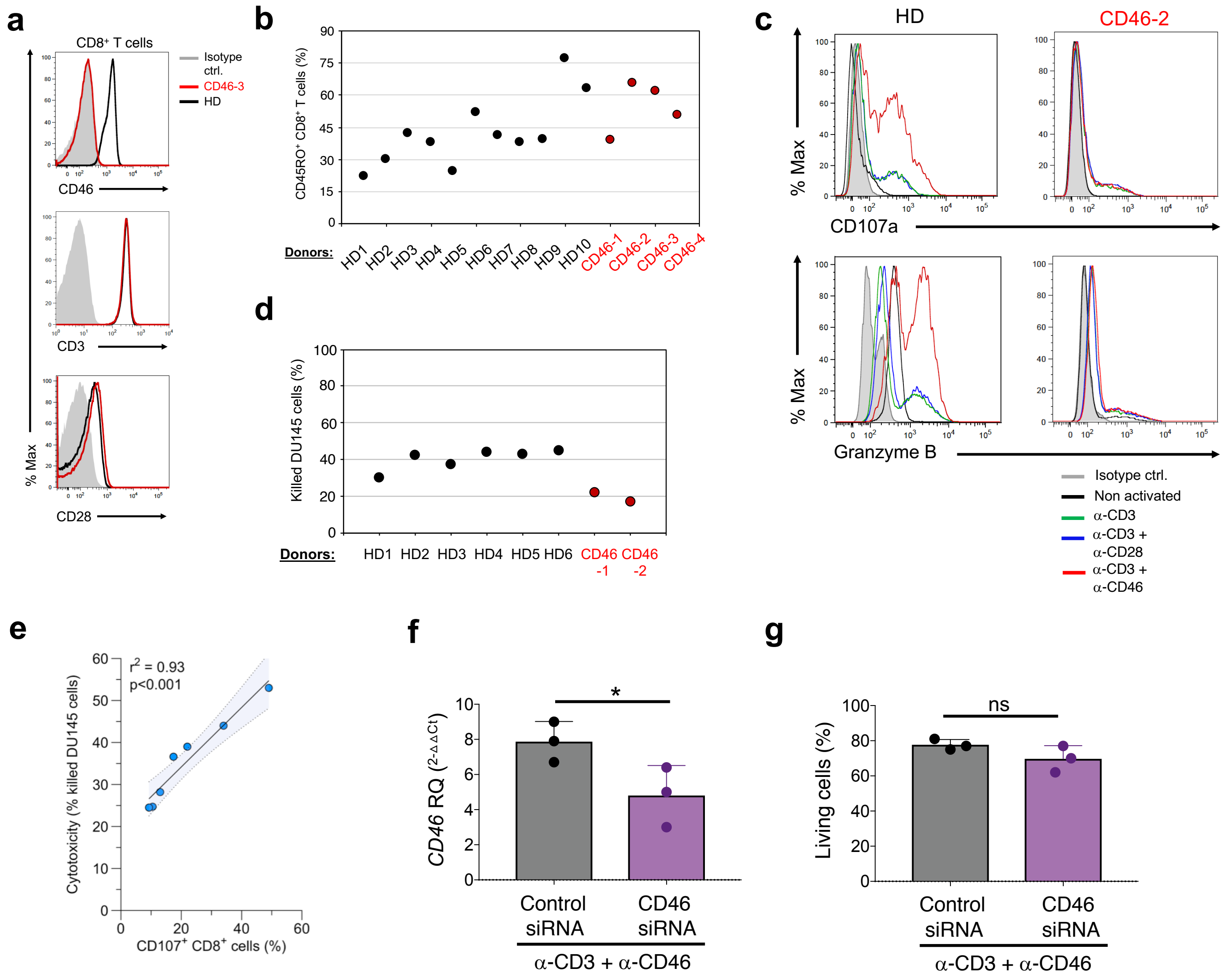

Supplementary Figure 3. CTL activity of CD46-deficient patients and CD46 knock-down and CTL viability. **a** CD46, CD3 and CD28 expression on CTLs (gated as in Supplementary Fig. 6b) from CD46-deficient patients and matched healthy donors (HDs) (grey histograms, Isotype control; black histograms, HD cells; red histograms, cells from patient CD46-3). **b** Proportion of circulating naive and memory CD8$^+$ T cells (gated as in Supplementary Fig. 7d) in the CD46-deficient patients in comparison with sex- and age-matched HDs. **c** CD107a and granzyme B expression by CTLs (gated as in Supplementary Fig. 7b) from patient CD46-2 at 60 hrs post activation under the indicated conditions (grey histogram, Isotype control; black histogram, non activated cells; green, blue, and red histograms, CD3, CD3+CD28, or CD3+CD46 activation, respectively) **d** Killing ability of CTLs from patients CD46-1 and -2 in comparison to HDs (gating strategy in Supplementary Fig. 7c). **e** Correlation between CD107a expression and cytotoxic activity in CD3+CD46-activated CTLs from HDs. **f** and **g** Effects of CD46 siRNA treatment of CD8$^+$ T cells on CD46 expression and viability. CD8$^+$ T cells were activated with antibodies to CD3+CD28 in the presence of either a siRNA targeting CD46 mRNA or a control siRNA for 48 h. CD46 mRNA levels were assessed by Q-PCR (**f**) and cells were then CD3+CD46 activated for 48 hrs and viability monitored (**g**) (n = 3). Error bars denote mean ± SEM. *, $p < 0.05$; ns, statistically not significant. Statistical analyses were performed using a simple regression analysis or the Paired Student's t-test.

Supplementary Figure 4

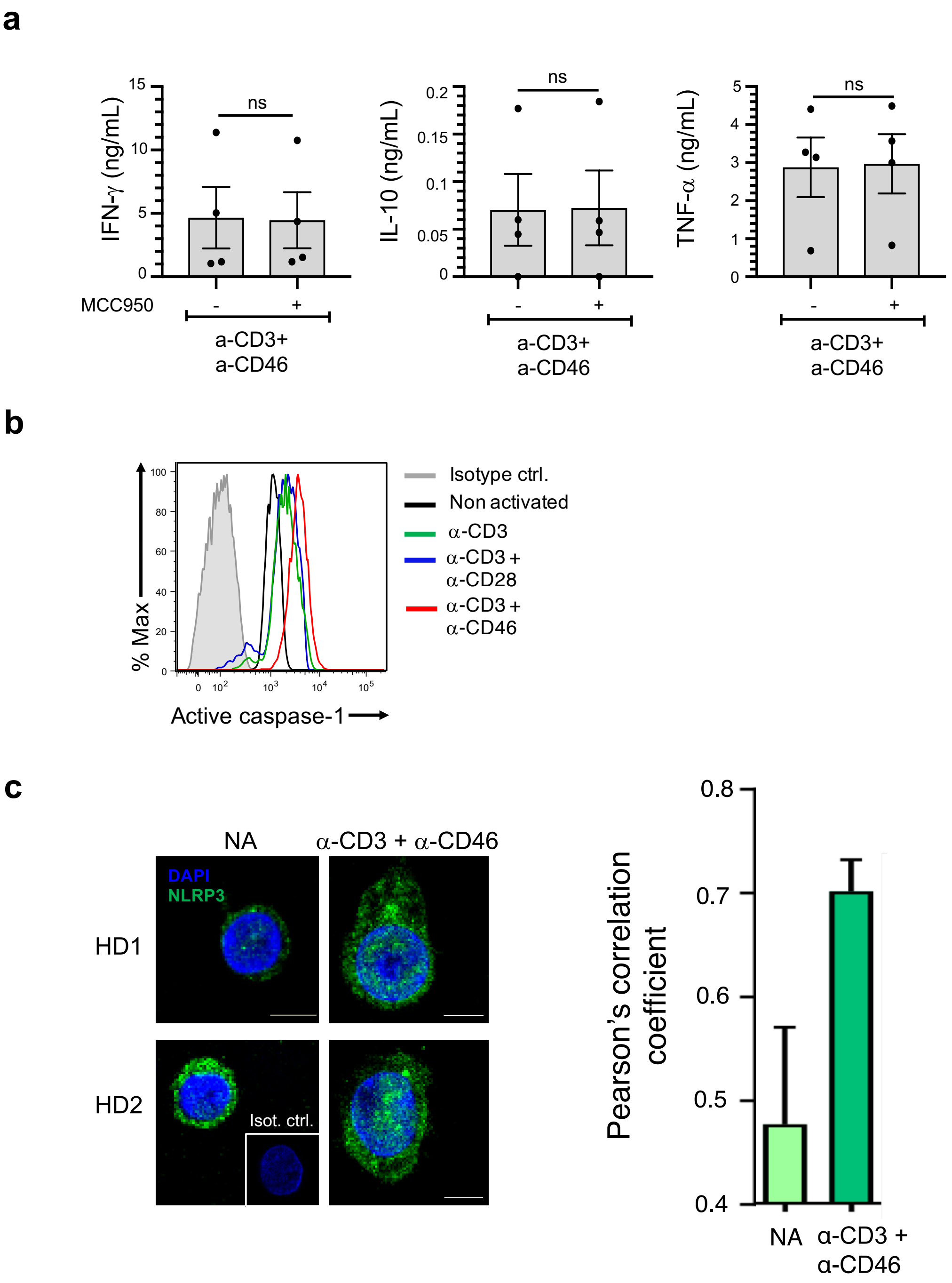

Supplementary Figure 4. Effect of MCC950 on human CD8+ T cell cytokine production. a Impact of MCC950 on IFN-γ, IL-10 and TNF-α production by CD46-activated CD8+ T cells. Purified T cells were activated with immobilized antibodies to CD3 and CD46 in the presence or absence of MCC950 (10 μM) and cytokine production determined at 60 h post activation (n = 4). **b** Representative plot for the FACS-based measurement of active caspase-1 using the FLICA assay (gating based as shown in Supplementary Figure 7b) (grey histogram, Isotype control; black histogram, non activated cells; green, blue, and red histograms, CD3, CD3+CD28, and CD3+CD46 activated cells, respectively). **c** NLRP3 protein translocates to the nucleus upon CD3+CD46 activation. CTls were activated as shown and the localization of NLRP3 monitored by confocal microscopy at 12 hrs post activation. The left panel shows a representative visualization of NLRP3 staining from one donor whilst the right panel shows the analysis of nuclear translocation measured by the Pearson's correlation coefficient (n = 3). The white size bar in the left panels denotes 5 μM. Error bars denote mean ± SEM. ns, statistically not significant. Statistical analyses were performed using the Paired Student's t-test.

Supplementary Figure 5

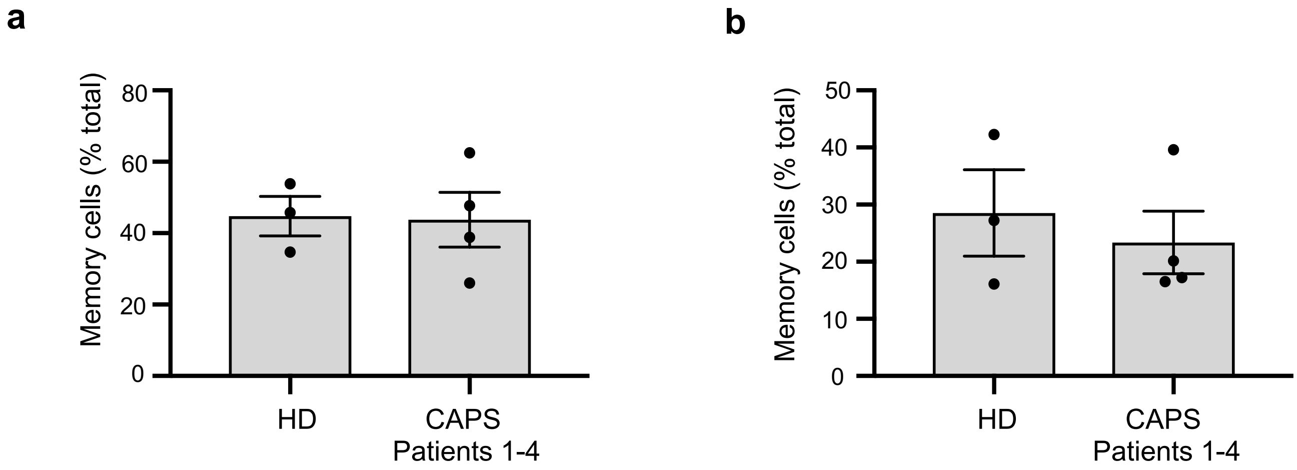

Supplementary Figure 5. CAPS patients naive and memory CD4^{+} and CD8^{+} T cells. Proportion of naive and memory CD4^{+} (**a**, cells gated as in Supplementary Fig. 7e) and CD8^{+} (**b**, cells gated as in Supplementary Fig. 6d) T cells of CAPS patients 1-4 in comparison to age- and sex-matched HDs 1-3 isolated T cells. Error bars denote mean ± SEM, statistical analyses were performed using the Unpaired Student's *t*-test and showed not significant differences among HDs and CAPS patients.

Supplementary Figure 6

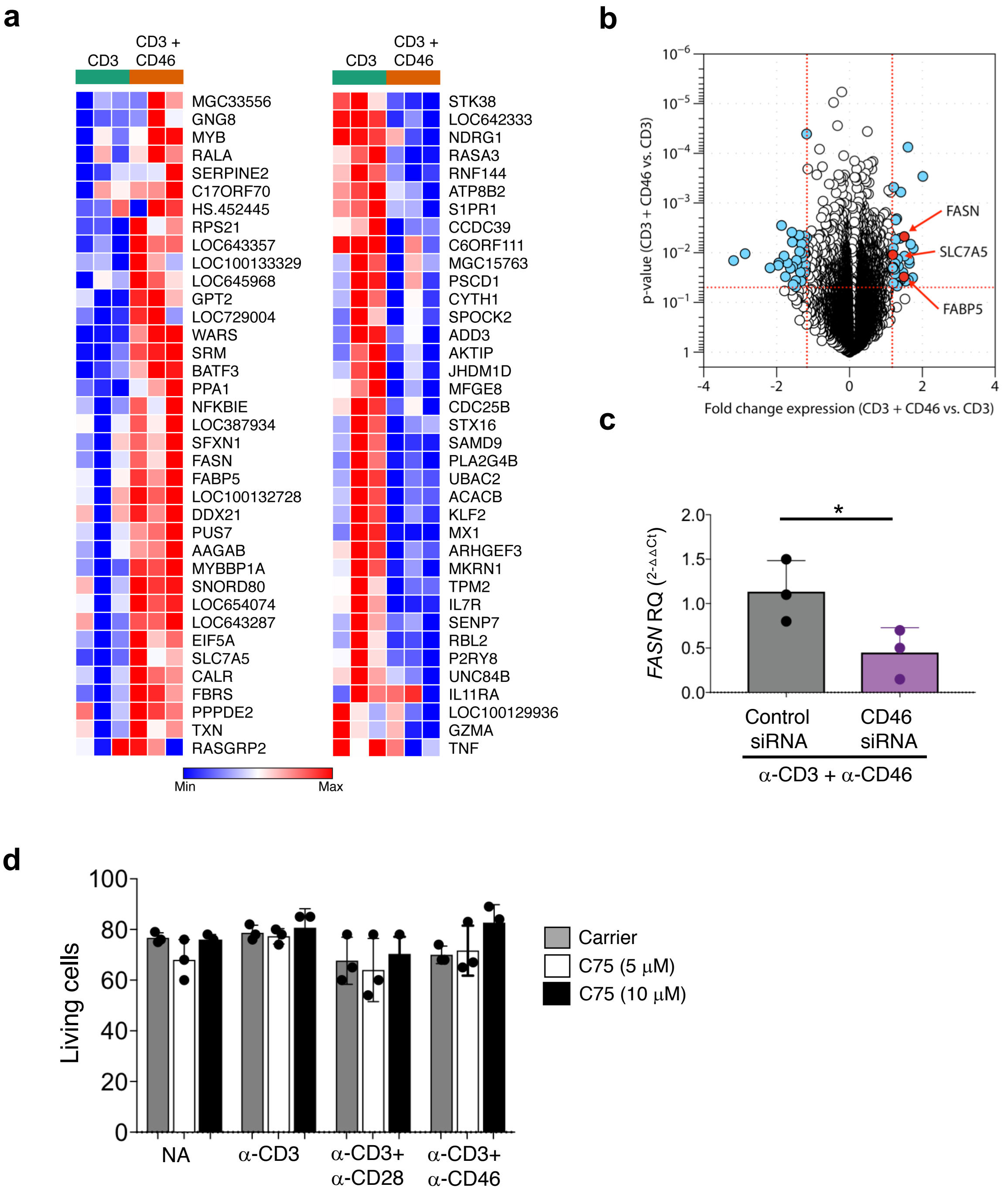

Supplementary Figure 6. CD46 co-stimulation augments nutrient influx and fatty acid synthesis. **a** Heat map depicting differentially expressed genes in CD8+ T cells from three healthy donors after activation with antibodies to either CD3 alone or to CD3+CD46 (6 h) ($n = 3$). **b** Volcano plot depicting the fold-change and p-values of key genes regulated by CD46 co-stimulation at 6 h post activation. **c** Quantitative RT-PCR assessing fatty acid synthase (*FASN)* mRNA in non-activated (NA) and CD3, CD3+CD28, or CD3+CD46 activated CD8+ T cells from healthy donors at 6 h post activation ($n = 3$). **d** Effect of pharmacological FASN inhibition on the viability of CD8+ T cells. CD8+ T cells were activated with the depicted antibody combinations in the presence or absence of the FASN inhibitor C75 and viability measured at 48 h post activation (grey bars, carrier treated cells; white bars, 5 μM C75; black bars, 10 μM C75) . Error bars denote mean ± SEM. *, $p < 0.05$. Statistical analyses were performed using the Paired Student's *t*-test.

Supplementary Figure 7

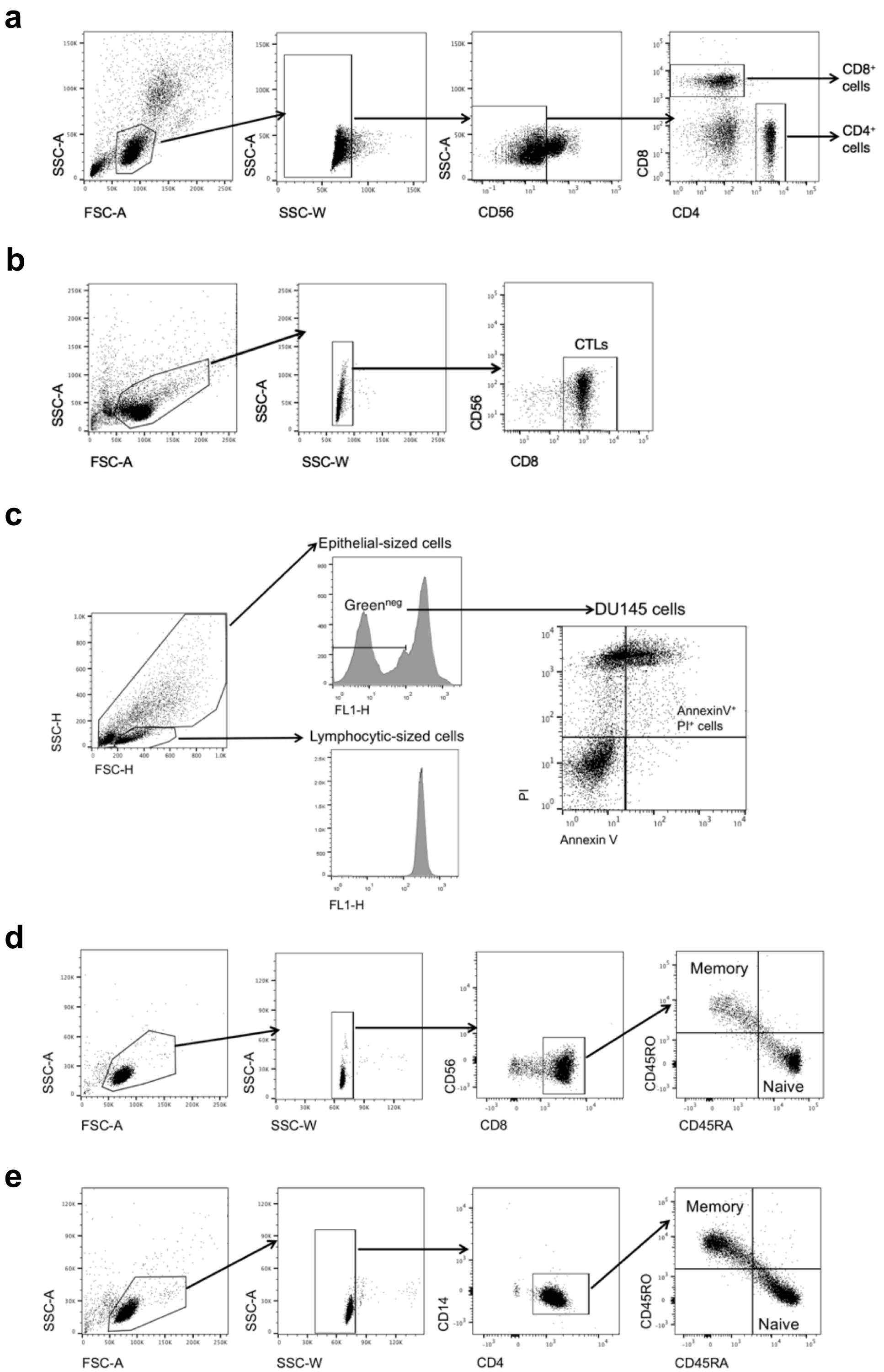

Supplementary Figure 7. Gating strategies used for flow cytometry stainings. a Gating strategy used to sort CD4$^+$ and CD8$^+$ T cells and for flow cytometry stainings presented on Figs. 1a, 3a, 4a-d and Supplementary Figs. 2a, 5b. **b** Gating strategy used for flow cytometry stainings on in vitro cultured sorted CD8$^+$ T cells presented in Figs. 1c-d, 2b,d, 3c,f,h, 4h-i, 5c, 6d-e and Supplementary Figs. 2b, 3a,c, 4b. **c** Gating strategy used for cytotoxicity assay of CD8$^+$ T cells co-cultured with DU145 cells presented in Figs. 1e, 3d, 5d and Supplementary Figs. 1c, 3d-e. The cancer epithelial cells were selected according to their bigger size, green positive CFSE-labelled T lymphocytes were excluded, and killing assessed by AnnexinV and PI (propidium iodide) staining. **d** Gating strategy used for sorting and flow cytometry stainings on naïve and memory CD8$^+$ T cells presented in Fig. 1g and Supplementary Figs. 3b, 5b. **e** Gating strategy used for flow cytometry staining on naïve and memory CD4$^+$ T cells presented in Supplementary Fig. 5a.

Supplementary Figure 8

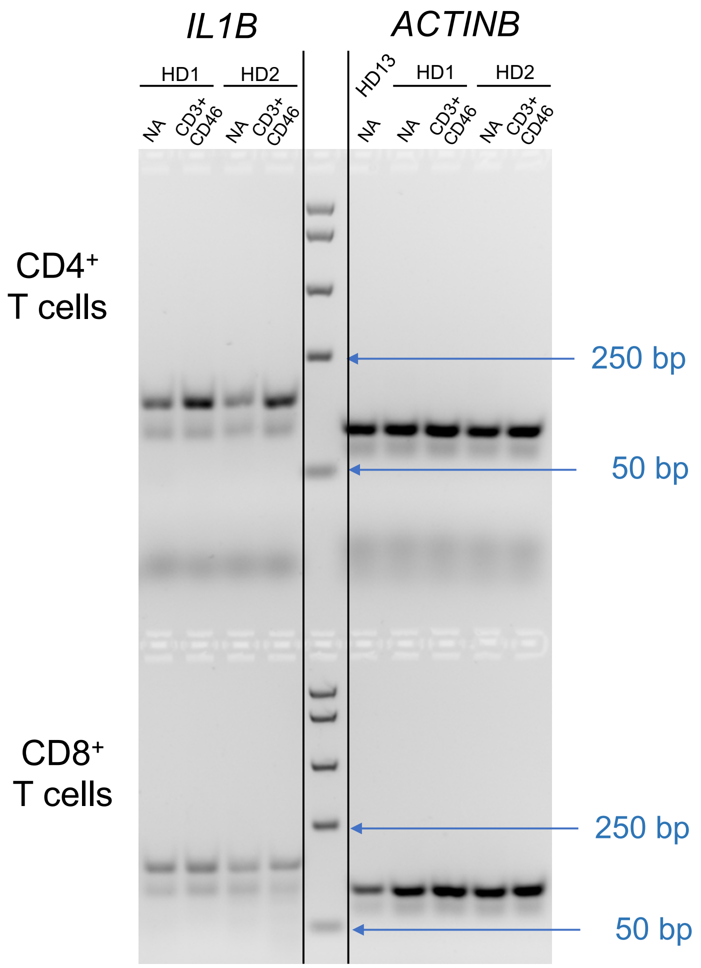

Supplementary Figure 8. Uncropped blot depicting *IL1B* message levels in CD4⁺ and CD8⁺ T cells. Original and uncropped blot showing the levels of *IL1B* and *ACTINB* mRNA in resting and CD3+CD46-activated CD4⁺ and CD8⁺ T cells from two healthy donors (60 hr post activation) corresponding to Figure 4e in the main manuscript. HD13 depicts an additional non-activated loading control used but not shown in Figure 4e.

SUPPLEMENTARY TABLES

Supplementary Table 1. Details of patients with CD46 deficiency that participated in this study

	**CD46-1**	**CD46-2**	**CD46-3**	**CD46-4**
**Gender**	Male	Male	Female	Female
**Age (years)**	36	29	39	31
**Ethnicity**	Cauc	Cauc	Cauc	Cauc
**Mutation**	Exon 2;<br>1. Missense Cys1Tyr<br><br>2. Nonsense Arg25X. X=stop<br><br>No detectable protein on T cells	Exon 2;<br>1. Splice site alteration IVS2+1G>C leading to three aberrant mRNA transcripts.	Exon 2;<br>1. Splice site alteration IVS2+2T>G leading to deletion of c144/p48 in frame with residual WT sequence.	Exon 2;<br>c.286+2T>G<br><br>Intronic;<br>c.1127+2T>G
**Homozygous Heterozygous**	Compound heterozygous	Homozygous	Homozygous	
**Hemolytic Uremic Syndrome**	+	+	+	+
**Reference**	Le Friec et al. Nat Immunol, 2012[1]	Couzi L. et al., Am J Kid Dis., 2008[2];<br>Le Friec et al. Nat Immunol, 2012[1]	Fremeaux-Bacchi V. et al., JASN 2006[3];<br>Le Friec et al. Nat Immunol, 2012[1]	Not published yet

**, Cauc, caucasion*

Supplementary Table 2. CD46, CD3 and CD28 expression on CTLs from healthy donors (HD) and CD46-deficicient patients 1-4.

	CD46 expr. (ΔMFI Isotype. ctrl.)	CD3 expr. (ΔMFI Isotype ctrl.)	CD28 expr. (ΔMFI Isotype ctrl.)
HD1/CD46-1	326/4	6433/6502	245/237
HD2/CD46-2	451/12	6178/6785	195/227
HD3/CD46-3	1096/4	5281/4659	215/226
HD4/CD46-4	76/0	242/292	16/13.5

Data are related to Supplementary Fig. 3A and represent values of variation in MFI (mean fluorescence intensity) of the marker stainings after substraction of respective isotype control.

Supplementary Table 3. Characteristics of patients with *NLRP3* mutations

Patient N^{o}	Age	Gender	NLRP3 Mutation
1	22	Female	V198M
2	53	Female	A439V
3	53	Male	A439V
4	30	Male	A439V

Supplementary Table 4. CTL activity of CTLs from healthy donors (HD) versus CAPS patients (P)

	CD107a$^+$ cells (%)	Granzyme B$^+$ cells (%)	Killing (%)
HD1	10.5	24.7	14.9
HD2	9.25	24.5	36.4
HD3	17.4	36.6	30.8
P1	12.2	43.5	41.3
P2	9.63	9.9	39.3
P3	12.8	31.7	40.4
P4	4.75	14.8	16.8

SUPPLEMENTARY REFERENCES

1. Le Friec, G. et al. The CD46-Jagged1 interaction is critical for human TH1 immunity. *Nat. Immunol*. 13, 1213–1221 (2012).

2. Couzi, L. et al. Inherited deficiency of membrane cofactor protein expression and varying manifestations of recurrent atypical hemolytic uremic syndrome in a sibling pair. *Am. J. Kidney Dis*. **52**, 5–9 (2008).

3. Fremeaux-Bacchi, F. et al. Genetic and functional analyses of membrane cofactor protein (CD46) mutations in atypical hemolytic uremic syndrome. *J. Am. Soc. Nephrol.* **17**, 2017–2025 (2006).
