# Supplementary material for: Complement receptor CD46 co-stimulates optimal human CD8+ T cell effector function via fatty acid metabolism
Source: Nat Commun. 2018 Oct 10;9:4186. doi: 10.1038/s41467-018-06706-z (PMC6180132; doi:10.1038/s41467-018-06706-z)
Supplement: Supplementary file 3 — Description of Additional Supplementary Files [file 41467_2018_6706_MOESM3_ESM.pdf]

SUPPLEMENTARY DATA

Complement receptor CD46 co-stimulates optimal human $CD8^+$ T cell effector function via fatty acid metabolism

Arbore et al.

Supplementary Data 1:
Excel File depicting the differentially expressed genes (DEGs) assessed by RNA-Seq between healthy Donor and CD46-deficient patient $CD8^+$ T cells 6 h post CD3+CD46 activation (related to Figure 6).

Supplementary Data 2:
Excel file showing enriched pathways based on pathway analysis of DEGs comparing healthy Donor and CD46-deficient patient $CD8^+$ T cells 6 h post CD3+CD46 activation (related to Figure 6).

Supplementary Data 3:
(DEGs) derived from the comparison of CD3 and CD3+CD46 activated $CD8^+$ T cells of healthy donors 6 h post activation (related to Figure 6).
